# The Utilization of Imaging Features in the Management of Intraductal Papillary Mucinous Neoplasms

**DOI:** 10.1155/2014/765451

**Published:** 2014-08-19

**Authors:** Stefano Palmucci, Claudia Trombatore, Pietro Valerio Foti, Letizia Antonella Mauro, Pietro Milone, Roberto Milazzotto, Rosalia Latino, Giacomo Bonanno, Giuseppe Petrillo, Antonio Di Cataldo

**Affiliations:** ^1^Radiodiagnostic and Radiotherapy Unit, University Hospital “Policlinico-Vittorio Emanuele”, Via Santa Sofia 78, 95123 Catania, Italy; ^2^Department of Surgical Sciences, Organ Transplantation and Advanced Technologies, University Hospital “Policlinico-Vittorio Emanuele”, 95123 Catania, Italy; ^3^Gastroenterology Unit, University Hospital “Policlinico-Vittorio Emanuele”, 95123 Catania, Italy

## Abstract

Intraductal papillary mucinous neoplasms (IPMNs) represent a group of cystic pancreatic neoplasms with large range of clinical behaviours, ranging from low-grade dysplasia or borderline lesions to invasive carcinomas. They can be grouped into lesions originating from the main pancreatic duct, main duct IPMNs (MD-IPMNs), and lesions which arise from secondary branches of parenchyma, denominated branch-duct IPMNs (BD-IPMNs). Management of these cystic lesions is essentially based on clinical and radiological features. The latter have been very well described in the last fifteen years, with many studies published in literature showing the main radiological features of IPMNs. Currently, the goal of imaging modalities is to identify “high-risk stigmata” or “worrisome feature” in the evaluation of pancreatic cysts. Marked dilatation of the main duct (>1 cm), large size (3–5 cm), and intramural nodules have been associated with increased risk of degeneration. BD-IPMNs could be observed as microcystic or macrocystic in appearance, with or without communication with main duct. Their imaging features are frequently overlapped with cystic neoplasms. The risk of progression for secondary IPMNs is lower, and subsequently an imaging based follow-up is very often proposed for these lesions.

## 1. Introduction

Intraductal papillary mucinous neoplasms (IPMNs) are a subgroup of cystic pancreatic neoplasms, representing an estimated 0.5–9.8% of all pancreatic exocrine tumours [1, 2]. Their incidence has been modified in the last decade, due to the large amount of IPMNs occasionally reported after cross-sectional imaging [3, 4]. In the last 15 years also Salvia et al. confirmed the increase in frequency of IPMNs, with an incidence of disease ranging again from 0.5% up to 10% among all exocrine pancreatic tumours [5–8].

Initially, it was difficult to define their nosological entity and, consequently, these mucinous ductal tumours were known variously [1, 9]. Only in 1997 the term intraductal papillary mucinous neoplasms (IPMN) was introduced by the WHO (Word Health Organization) [1, 10]. The term refers to a group of pancreatic neoplasms originating—in papillary form—from the epithelium of the duct system and leading progressively to a dilatation of the duct, which progressively develops a cystic appearance.

As with many other cancers, the origin of IPMNs is still unknown. Since they were first reported, they have been associated with chronic pancreatitis.

In a recent multicentre control-case study published, some clinical conditions have been associated with the development of IPMN, including diabetes (particularly cases associated with insulin assumption), chronic pancreatitis, and a family history of pancreatic ductal adenocarcinoma [11].

The natural history of small pancreatic cysts is not yet clearly understood. According to their biological behaviour, the WHO classification system currently separates IPMNs into

- benign (intraductal papillary mucinous adenoma)
- borderline (intraductal papillary mucinous tumors with moderate dysplasia)
- malignant (intraductal papillary mucinous carcinoma, noninvasive or invasive).

In fact, IPMNs display a spectrum of cytoarchitectural atypia, ranging from none to borderline to marked and can also be associated with invasive carcinoma [12]. Similarly to the mucinous cystic neoplasms and the pancreatic intraepithelial neoplasia (PanIN), IPMNs are currently considered precursors and precancerotic lesions of the pancreas [13]. The transformation from a benign into a malignant histologic type may take several years (approximately 5 years) and this event is not observed in all cases [14].

Cystic pancreatic neoplasms include a large spectrum of lesions with different radiological appearance [1, 3, 15–18]. Their diagnosis requires a multidisciplinary approach [19, 20] because a significant overlap of clinical and radiological features has been reported among these tumours. The knowledge of typical imaging features of IPMNs is crucial for making a correct diagnosis, excluding not only other pancreatic cystic lesions but also peripancreatic structures which could simulate pancreatic disease [21].

Indeed, the aim of this review is to describe the imaging features of IPMNs, emphasizing the most important signs involved in the management of these neoplasms.

## 2. Cross-Sectional Imaging Features

IPMNs can develop at any point in the pancreatic ductal system. According to their site of origin, they are distinguished into [9]

- main duct IPMNs (MD-IPMNs)
- branch-duct IPMNs (BD-IPMNs)
- both (mixed type).

Main radiological features of IPMNs have been reported in a popular pictorial essay by Procacci et al. in 1999 [9].

**Figure 1:**
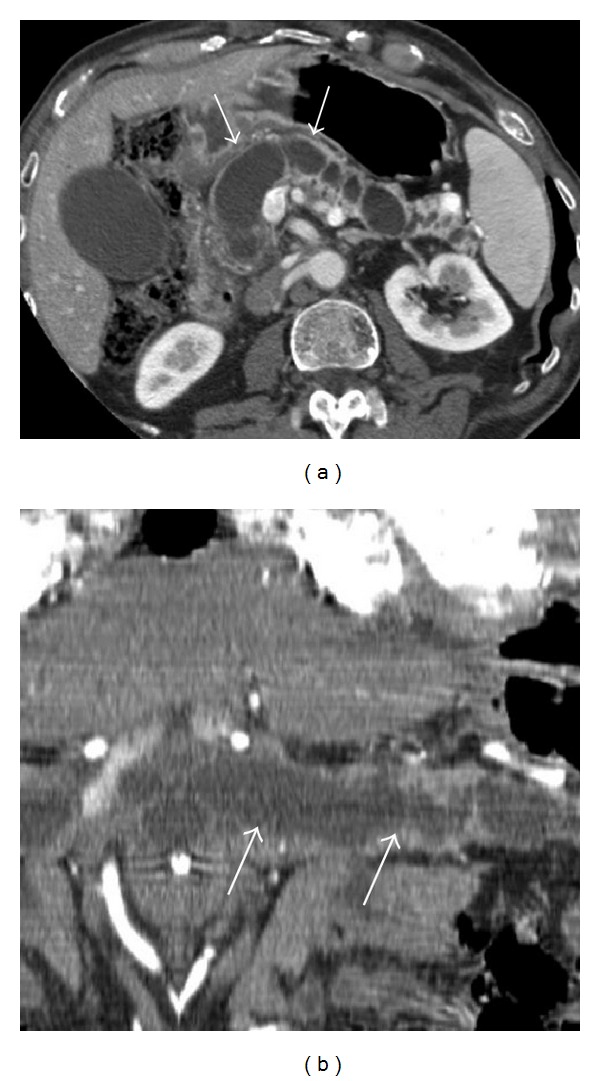
CT postcontrast examination in a patient who was suffering from jaundice and abdominal pain: axial images after contrast administration (a) and curved-MPR images (b). White arrows in (a) and (b) show a marked dilatation of the entire main pancreatic duct, from the head to the tail of the gland, associated with subtotal parenchymal atrophy. No dilatation of secondary branches was observed, and radiological diagnosis of MD-IPMN was formulated. The high degree of main pancreatic duct dilatation (>1 cm) was considered as high-risk stigmata and required surgical treatment. In addition, white arrows show mild wall enhancement. Final diagnosis of invasive cancer (adenocarcinoma) in IPMN was reported.

**Figure 2:**
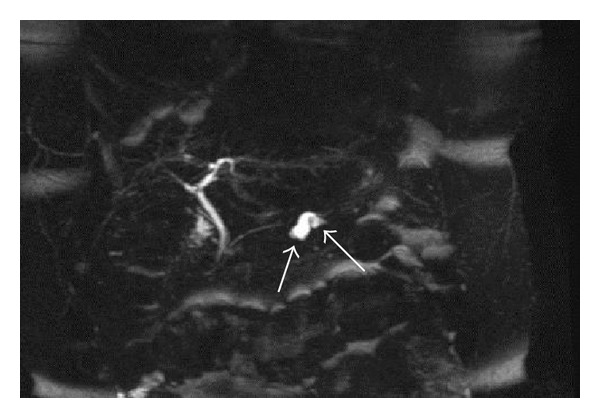
BD-IPMN in a 67-year-old female. MRCP acquisition clearly shows a cystic lesion centred on the body of pancreatic parenchyma (white arrows). The cyst shows a curved tubular shape. Due to the absence of high-risk-stigmata and worrisome features, lesions were safely managed.

MD-IPMNs originate from the main pancreatic duct and are also indicated as* “Primary IPMNs”* (Figure 1). They may exhibit a diffuse or segmental involvement of main pancreatic duct. BD-IPMNs, which develop from secondary branches of main pancreatic duct, have been also reported as* “Secondary IPMNs”* (Figure 2).

Several studies have documented the different biological behaviour of primary and secondary IPMNs. The possibility of malignant degeneration is strongly dependent on the site of origin because MD-IPMNs show a risk of progression of 60–92%, whereas IPMNs arising from secondary branches have a lower value of degeneration, approximately 6–40% [22, 23].

Mixed type includes a combined pattern of presentation, with involvement of both main pancreatic duct and secondary branches.

A recent “*European experts consensus statement on cystic tumours of the pancreas*” [24] clearly suggests that the main role of CT/MR imaging is “*to reduce differential diagnoses when a cystic pancreatic lesion is revealed by ultrasonography*.” Thus, MR and MRCP play an important role in the identification of the relationship between cystic lesions and pancreatic duct system. In case of connection, a diagnosis of IPMN could be suggested [24], whereas when connection is not identified, alternative diagnoses should include serous cystadenoma or mucinous cystadenoma. These cystic neoplasms are differentiated on the basis of their architecture: honeycombing and microcystic appearance are generally associated with serous lesions, whereas oligocystic/macrocystic appearance is frequently encountered in cases of mucinous cystadenomas [3, 16, 24, 25]. In addition, site of lesion and gender are important factors used for differential diagnosis [25].

Currently, cross-sectional imaging modalities have high accuracy in the diagnosis and assessment of loco-regional infiltration of cystic tumours of the pancreas; namely, CT has accuracy of 1.2–2.9%, whereas MRI reports higher values — 13.5–44.7% [24].

**Figure 3:**
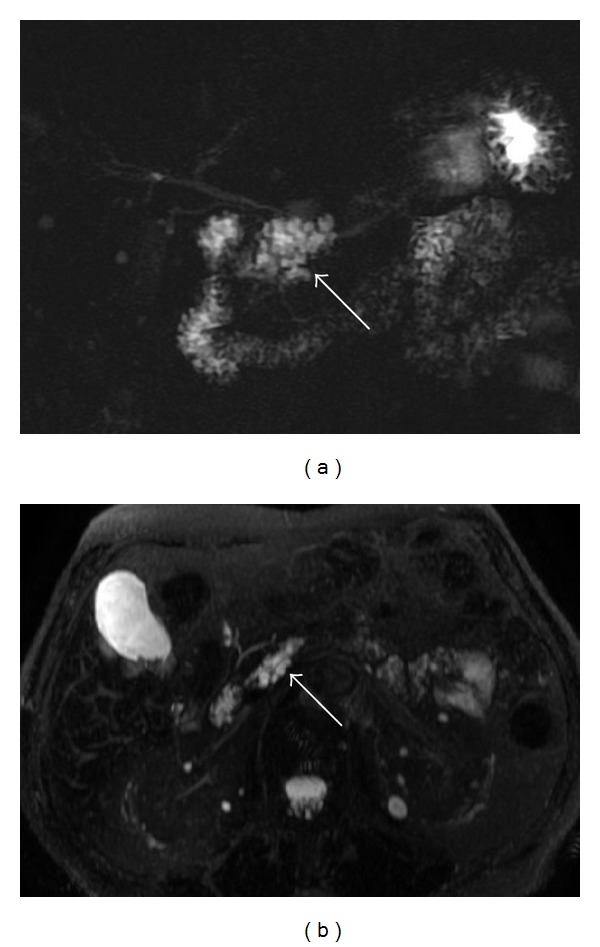
MRCP images (a and b), obtained using 2D FSE sequence and 3D FRFSE technique, respectively. BD-IPMN of about 3 centimeters located in the uncinate process of pancreas, with a typical microcystic appearance. No other worrisome features were found by EUS; the patient was successfully enrolled in a follow-up program.

MRI and MRCP clearly distinguish the cystic dilatation of main pancreatic duct due to their high contrast resolution. Two-dimensional single shot fast spin echo (SSFSE) sequences and three-dimensional (3D) fast recovery fast spin echo (FRFSE) sequences are generally able to demonstrate the dilatation of main pancreatic duct or the cystic lesion originating from main duct (Figure 2). 3D FRFSE sequences may recognize the dilatation of main pancreatic duct also using multiplanar reconstruction (MPR) or maximum intensity projection (MIP) postprocessing techniques (Figure 3) [24, 26].

MPR images are strongly recommended for the identification of the communication of secondary IPMNs with main pancreatic duct. In a recent study by Sahani et al. [27] CT and MRCP were compared in the assessment of BD-IPMNs. For cyst communication, the overall sensitivity values of multidetector CT and MRCP were, respectively, 83% and 87%. Due to their high diagnostic performance, MPR/MIP postprocessing need to be performed simultaneously during CT and MR/MRCP examinations [24].

**Figure 4:**
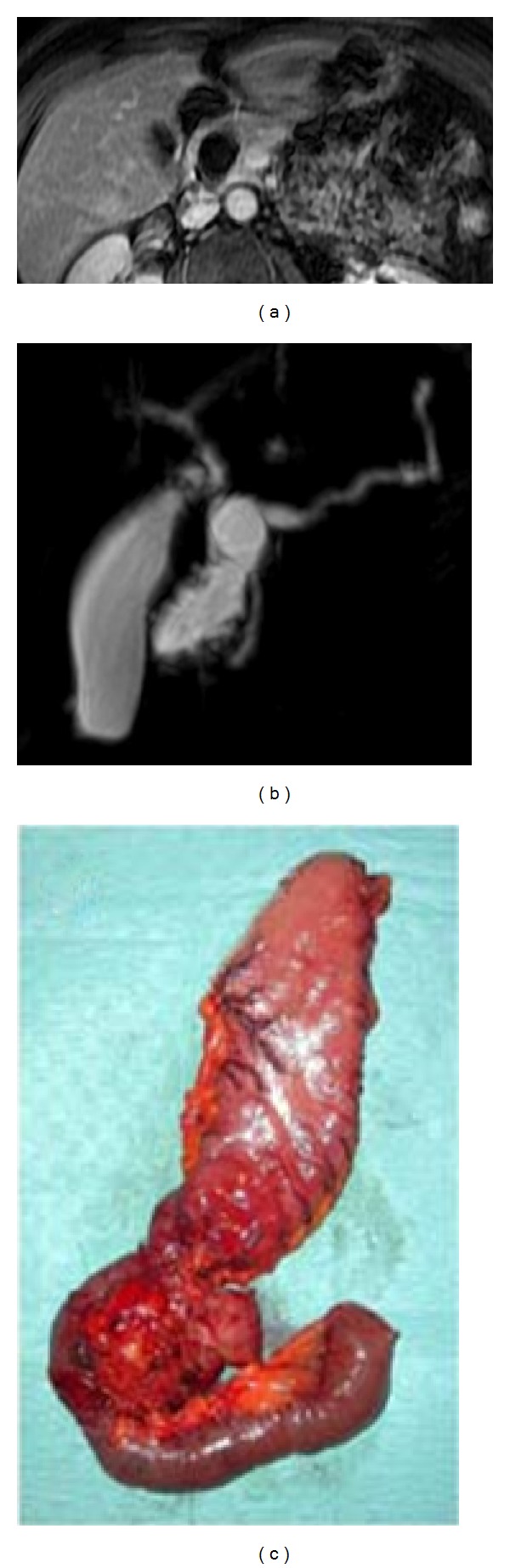
Axial T1-weighted spoiled gradient echo after gadolinium administration (a). 3D FRFSE MRCP sequence obtained using MIP reconstruction (b). Surgical specimen (c), from poster EPOS C-2228 presented in [15]. (a) shows a homogeneous cystic lesion centered in the head of pancreas. No intralesion solid components were observed. In (b), MIP reconstruction was useful to better appreciate the cystic morphology of the lesion due to main pancreatic duct enlargement. Again, high-risk stigmata (main duct caliber >1 centimeter) suggested surgical management. A pancreatoduodenectomy was performed and final diagnosis deposed for borderline IPMN.

The goal of both cross-sectional imaging modalities—CT and MR with MRCP—is to identify some imaging features reported as “high-risk stigmata” or “worrisome feature” in the evaluation of pancreatic cysts. “High-risk stigmata” include essentially main pancreatic duct dilatation ≥10 mm (Figures 1 and 4) and the presence of solid components showing enhancement after contrast administration [28].

“Worrisome features,” reported by IAP, are size of cyst ≥3 cm, thickened cyst wall with enhancement after contrast administration, mural nodules without enhancement after contrast, main duct with diameter of 5–9 mm, abrupt change in the main pancreatic duct caliber with distal pancreatic atrophy, and lymphadenopathy [28].

### 2.1. MD-IPMNs

MD-IPMNs are usually located in the proximal portion of the gland (75%), but they can also be recognized in the rest of the pancreatic parenchyma [29]. Main pancreatic duct dilatation is the typical radiological feature observed in primary IPMNs, involving the full length of the duct; segmental or diffuse dilatation of main pancreatic duct should exceed 5 mm, even if recent articles report that a lower size (5 mm) could be also adopted for the diagnosis of MD-IPMNs [28].

The measurement of main pancreatic duct is a crucial step in the evaluation of MD-IPMNs: a diameter of 5–9 mm is considered a “worrisome feature,” whereas main duct measurement ≥10 mm is reported as “high-risk stigmata.”

Both CT and MRI images could demonstrate the increased size of the duct, as its progressive dilatation could induce a parenchymal atrophy (Figure 1). Another typical finding observed in MD-IPMNs is the dilatation of the major papilla, the minor papilla, or both, with a bulging of the main pancreatic duct into the duodenal lumen [30]. Moreover, the diffuse main pancreatic duct dilatation is often associated with the dilatation of some branch ducts, particularly in the uncinate process and in the tail of the pancreas.

Both diffuse and segmental primary IPMNs have been associated with malignancy in the case of mural nodules or internal solid components [3–5, 22, 29]. The presence of solid components with enhancement after contrast administration has been reported as “high-risk stigmata” [28]. For this reason, CT and/or MRI examinations with contrast administration are recommended to better assess enhancement of internal nodules in primary IPMNs.

The diagnosis of IPMNs with a segmental involvement of the main pancreatic duct may be difficult because segmental dilatation rarely evolves into the cystic appearance (Figure 4). If the lesion is localized in the body or in the tail of the pancreas, the remainder of gland is normal. When lesion is located in the pancreatic head, it is often associated with upstream dilatation of the main pancreatic duct [9].

Primary IPMNs with cystic appearance require a differential diagnosis from mucinous cystadenoma. The dilatation of main pancreatic duct is generally observed in cystic IPMNs, whereas mucinous cystadenoma is rarely associated with main duct dilatation [9].

Primary IPMNs should be differentiated from chronic pancreatitis. Kim et al. investigated main radiological features which could be helpful for the differential diagnosis. These features include “duct dilatation without stricture, bulging ampulla, nodule in a duct, a grape-like cyst shape, and nodule in a cyst” [30].

The presence of internal nodules is more frequently associated with IPMNs than with pancreatitis. MRCP images clearly depict nodules and papillary projections, which usually appear as filling defects within the cystic lesions. However, in chronic pancreatitis ductal calcifications could simulate solid components, with hypointense signal on T2-weighted images. CT scan is able to demonstrate calcifications and help radiologists in the differential diagnosis between the two clinical entities. In addition, as reported by Kim et al., the presence of stone is considered one of the most specific signs of chronic pancreatitis [30].

### 2.2. BD-IPMNs or “Secondary IPMNs”

BD-IPMNs or* “secondary IPMNs”* (Figure 2) appear as cystic masses and therefore their demonstration is easier than MD-IPMNs. The most involved pancreatic region is the uncinate process (Figure 3). Lesions can be arranged in a microcystic or macrocystic pattern.

The microcystic pattern is characterized by small cystic lacunae separated by thin septa. This aspect is similar to that of serous cystadenoma and only the demonstration of a communication between the lesion and the main duct permits a correct diagnosis.

The macrocystic pattern is the most frequent. Lesions show a unilocular or multilocular architecture. The demonstration of the communication with the main pancreatic duct is a sign of differentiation from other cystic lesions such as the mucinous cystadenoma. However, the communication with main duct is often not appreciable on MR images [3, 22].

Thickness and irregularity of the tumor wall and of the septa are variable and increase with malignancy. Namely, increased thickness of cyst wall, showing enhancement after contrast administration, and/or mural nodules without contrast enhancement represent worrisome features that radiologists should always include in their report [28]. Other worrisome features that have to be considered are cyst size exceeding 3 cm and main pancreatic duct caliber of 5–9 mm [28].

Other imaging features have to be considered before making a differential diagnosis. Mucinous cystadenoma may exhibit peripheral calcifications, which could reproduce an “eggshell” appearance [31]. Also, favourite locations in the pancreatic parenchyma are different for the lesions because secondary IPMNs are very often reported in the uncinate process [29], whereas mucinous cystadenoma is generally encountered in the body or in the tail of the pancreas.

IPMNs need to be differentiated from pancreatic pseudocysts, which develop as a complication of pancreatitis in up to 20–40% of cases [30]. In a recent work, “a grape-like appearance” has been associated with IPMNs in 79% of cases, whereas a unilocular cyst shape was reported in 34% of patients affected by chronic pancreatitis. However, unilocular secondary IPMNs are very difficult to differentiate from pseudocysts. Careful collection of clinical history is very important in these cases because pseudocysts generally develop as a complication of a severe episode of pancreatitis.

BD-IPMNs could be observed in a multifocal appearance. In this pattern of morphological presentation, IPMNs are divided into five classes: diffuse, proximal, proximally diffuse, distal, and bridge morphology [22]. The multifocality of IPMNs is responsible for an increased cumulative risk of neoplastic degeneration [32]. In this case, patients need to be followed over time in order to identify early signs of progression or degeneration.

### 2.3. EUS

Endoscopic ultrasonography (EUS) plays an important role in the diagnostic evaluation of IPMNs due to the possibility to collect fluid from cystic lesions. It can provide high resolution contrast images of pancreatic cystic lesions, demonstrating many important details about cystic lesions, such as wall thickness, presence of septa, and mural nodules [33]. In addition, it permits measurement of the pancreatic ducts and provides visualization of communication between cystic lesions and main pancreatic duct. Also strictures could be visualized along the course of main duct, contributing to the differential diagnosis between chronic pancreatitis and MD-IPMN [34–36].

**Figure 5:**
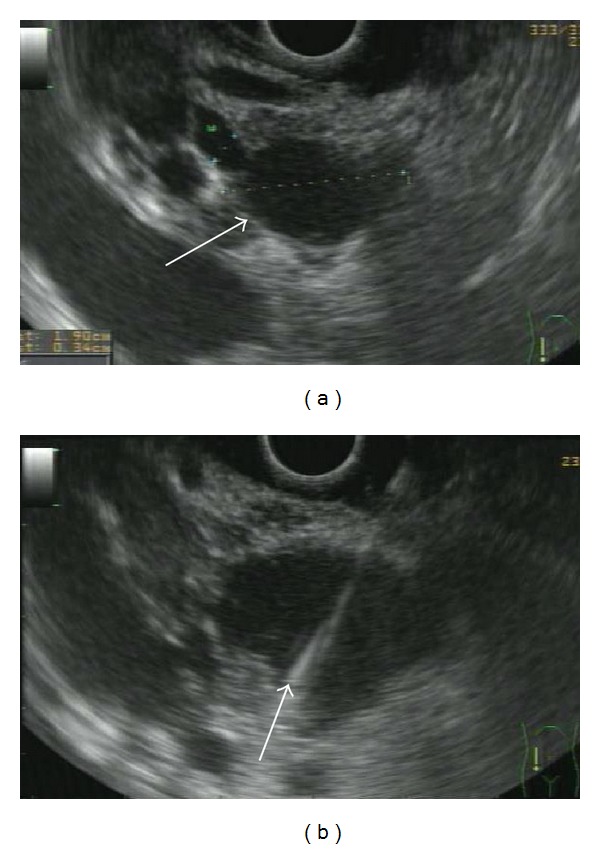
Linear EUS image of a MD-IPMN (a): a lobulated anechoic cystic lesion is clearly depicted (white arrow). (b) shows EUS-FNA of the same lesions. In this lesion (about 3 cm in size), the absence of mural nodules and positive or suspicious cytology allowed a conservative management.

In addition, EUS is able to guide fine-needle aspiration (FNA) (Figure 5) [37]. The fluid content could be analysed for the presence of oncological marker.

It has been well documented that CEA and CA 72.4 levels in the cystic fluid of the mucinous lesions are much higher (typically over 800 ng/mL) than those of nonmucinous ones [38]. Moreover, CEA and CA72.4 levels are higher in malignant mucinous neoplasms [39–43]. In a work by Brugge et al. a level of 192 ng/mL for CEA has a diagnostic sensitivity of 75%, a specificity of 84%, and an accuracy of 79% in differential diagnosis of mucinous and nonmucinous cysts [41].

In view of these considerations, several studies have recently investigated the diagnostic and prognostic values of these markers in order to establish the risk of malignant degeneration. Also inflammatory mediator proteins (cytokines, chemokines, and growth factors) — contained in pancreatic cyst fluid — could be used as potential diagnostic biomarkers able to characterize IPMNs [44]. However, sensitivity and specificity observed are not so high; detection of K-ras mutation in the pancreatic fluid can indicate the presence of a malignant cystic lesion, even with poor sensitivity (20%) [29]. The reported threshold level of 192 ng/mL for CEA has been evaluated as a predictor value of malignancy for IPMNs in a recent work by Kucera et al. [45]. The authors found that the mean level of intracystic CEA increases progressively from low-grade to high-grade of dysplasia (ranging from 1.261 ± 1.679 ng/mL to 10.807 ± 36.203 ng/mL). Among invasive cancers, the mean level reported was lower than IPMNs with various degrees of dysplasia. The reported sensitivity, specificity, positive predictive value, negative predictive value, and accuracy of a cyst fluid CEA concentration greater than 200 ng/mL for the diagnosis of malignant IPMN—including lesions with high-grade dysplasia and invasive IPMNs—were, respectively, 52.4%, 42.3%, 42.3%, 52.4%, and 46.8% [45].

On the basis of the mentioned studies, EUS—even with FNA—does not show such high values of sensitivity and specificity in the diagnosis of IPMNs. In addition, it is an invasive [24], heavily operator-dependent modality that requires patient sedation [37]. Recent* “European expert consensus statement on cystic tumours of the pancreas”* remarked that EUS is* “an invasive diagnostic procedure*,*”* which needs to be performed after cross-sectional imaging (CT/MRI), in a multimodality imaging assessment of cystic pancreatic neoplasms [24].

After CT/MRI examinations, “All cysts with worrisome feature or cysts exceeding 3 cm in size without worrisome feature” should be investigated by EUS [28]; identification of mural nodules, main duct signs of involvement by disease, or a cytology suspicion could suggest surgery [28].

Recently, some authors have proposed EUS imaging in the follow-up evaluation of secondary IPMNs. Kamata, in a recent retrospective study, compared the diagnostic value of EUS, ultrasonography, CT, and MRI in the assessment of pancreatic ductal adenocarcinoma arising from MD-IPMNs [46]. The population study included a total of 169 patients. All the mentioned imaging modalities followed 102 patients having side branch IPMNs without mural nodules and symptoms. The follow-up was performed in order to verify the incidence of IPMN-derived and/or concomitant pancreatic ductal adenocarcinoma. At the first follow-up examination, 17 IPMN-derived and 11 concomitant ductal adenocarcinomas were detected by the authors, with EUS overall sensitivity higher than other imaging modalities. For the entire follow-up period of the study, EUS maintained its better diagnostic accuracy in the detection of concomitant duct adenocarcinoma. Other authors have performed a follow-up study through US and MRCP in a large series of patients (*n* = 109) with BD-IPMNs [29]. In this study, EUS and ERCP were performed only in select cases, when the diagnosis was still unclear or doubtful after conventional cross-sectional imaging modalities.

However, the invasiveness and the variability represent limitations to adopting EUS in the follow-up of MD-IPMNs.

## 3. Management

Currently, management of IPMNs is one of the most debated topics in literature, and it is essentially based on cross-sectional imaging modalities (CT/MR) and EUS. There is no sufficient evidence for pancreatoscopy in management of cystic tumours and subsequently for IPMNs [24]. ERCP could be useful in selected cases, for example, in the evaluation of primary IPMNs with diffuse dilatation of main pancreatic duct, without evidence of mural nodules. In these cases, the diffuse increased caliber of main duct with bulging of major papilla promotes the right diagnosis of MD-IPMNs and could suggest the correct surgical approach.

First of all, cross-sectional imaging modalities should be able to clearly distinguish the three radiological patterns of presentation. As previously reported, primary IPMNs show a progression risk higher than secondary forms. In addition, multifocal branch-duct IPMNs have a cumulative risk of malignancy degeneration due to the coexistence of many cystic lesions.

High-risk stigmata, represented by dilatation of the main pancreatic duct equal to or more than 10 mm and/or solid components with enhancement after contrast, in view of its frequent association with malignancy, require surgical treatment. In fact, in a study performed by Abdeljawad, the prevalence of malignancy in 52 patients with pure main duct IPMN was analysed [47]. Among 16 asymptomatic patients reporting IPMNs, 4 had malignant lesions. In the symptomatic group (36 out of 52 patients), 25 lesions were malignant on histology. The size of the main pancreatic duct was analysed by authors using ROC analysis, and the largest area under the curve used to distinguish between benign and malignant MD-IPMN was found using a threshold level of main pancreatic duct of 8 mm (0.83; 95% CI = 0.72–0.94).

Worrisome features—including cyst size ≥3 cm, thickened cyst wall with enhancement after contrast administration, mural nodules without enhancement after contrast, main duct with diameter of 5–9 mm, and abrupt change in the main pancreatic duct caliber with distal pancreatic atrophy and lymphadenopathy—require further investigation [28]. As previously reported, EUS plays an important role in the management because confirmation of worrisome features could require a surgical treatment [28]. If absent, IPMNs could be monitored using MR/MRCP at 3 months and EUS annually for the first 2 years [28].

Regarding the size, cysts exceeding 3 cm, even if considered a worrisome feature, did not show a high value of correlation with malignancy. In a series observed by Sahani et al., only 5 out of 8 lesions with diameter >3 cm were malignant at pathological examination. In another series of 26 patients with secondary IPMNs reported by Manfredi et al., a significant change in the size of cystic lesions was observed. However, this imaging finding does not necessarily correlate with malignant transformation or increased suspicion of malignancy [4].

Therefore, the presence of nodules is probably the most significant change which needs to be carefully evaluated because it is strongly suspected as an indicator of malignancy.

Salvia has evaluated nonoperative management of secondary branches IPMNs in a prospective study, by performing contrast enhanced US and MRCP. Lesions were less than 3.5 cm in diameter and without nodules or solid components. Their study included a total of 109 patients. A first group (20 patients, 18.3%) required immediate surgery for the presence of symptoms or clinical and morphological features associated with malignancy. Among this group, the authors found only 2 patients with invasive carcinoma and 1 patient with carcinoma in situ. The remainder of the patients were evaluated with an average follow-up of 32 months. After an average follow-up of 18.2 months, Salvia et al. [29] reported only 5 patients with an increase in the size of the lesion. These patients underwent surgery and their final diagnosis was branch-duct adenoma in 3 cases and borderline lesions in 2 patients [29]. Thus, this study confirms that BD-IPMNs could be managed by imaging.

**Figure 6:**
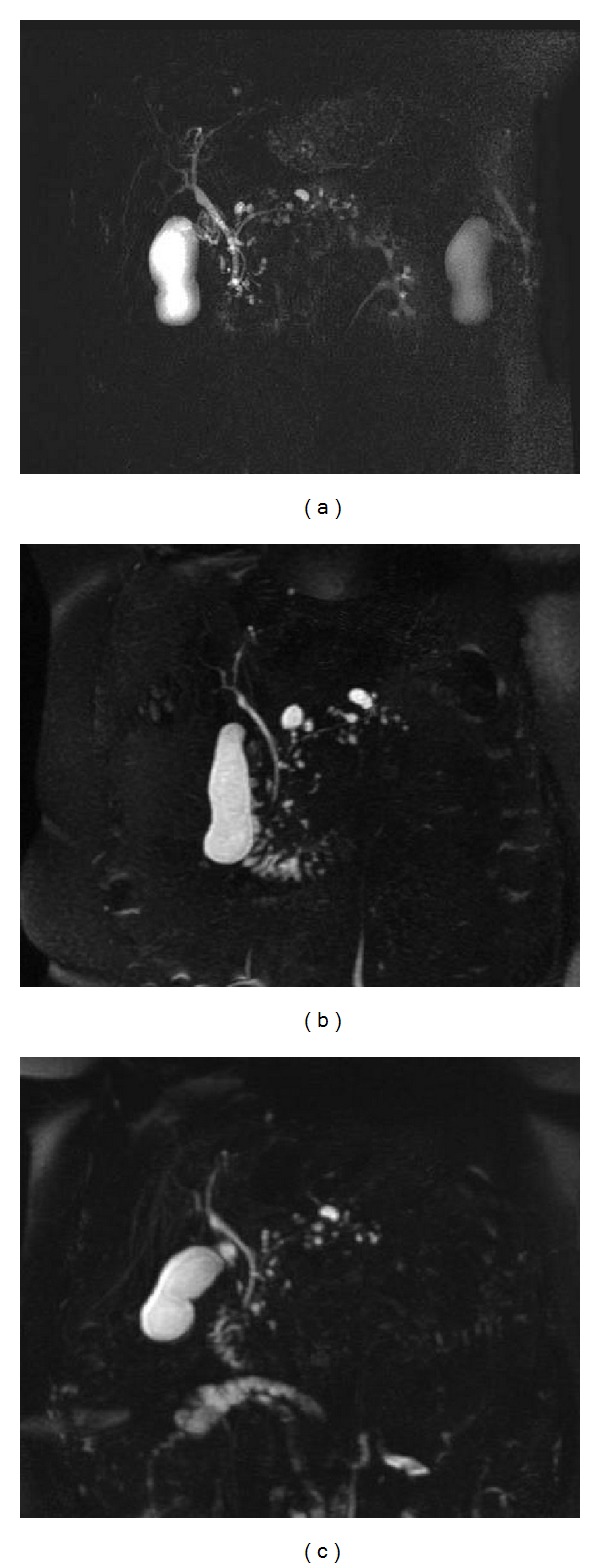
Coronal MRCP acquisitions in an asymptomatic 70-year-old female patient with an incidental radiological finding of multiple pancreatic cystic lesions; MRCP exams were performed in 2009 (a), in 2012 (b), and in 2013 (c). Multiple small cystic lesions in the pancreatic parenchyma are clearly depicted in the three MRCP acquisitions, some of them showing a typical connection to the main pancreatic duct. This typical radiological pattern suggests the diagnosis of multifocal BD-IPMNs. No main pancreatic duct dilatation is observed. Cystic lesions do not show intraluminal solid components or mural nodules. Over time the MRI monitoring initially showed a mild enlargement of the lesions (from a to b) and then a size-reduction (from b to c). As reported in literature, IPMNs in a multifocal setting could also be managed in a safe and reliable mode.

Finally, secondary IPMNs arranged in a multifocal pattern (Figure 6) should be evaluated for their increased risk of degeneration [48]. However, in another study, Salvia examined a total of 131 patients having multifocal secondary IPMNs. Here, only 10 patients were surgically managed, whereas the majority was followed for an average period of 40 months. 121 patients were conservatively managed, and they remained asymptomatic, without nodules or increase in diameter of their lesions. As reported by the authors, IPMNs in a multifocality setting could also be managed in a safe and reliable way [49].

## 4. Conclusion

Gastroenterologists, radiologists, and surgeons should be confident utilizing all imaging features of IPMNs.

On the basis of the diagnostic patterns analysed,

- radiologists should distinguish between primary, secondary, and mixed IPMNs; cross-sectional imaging features need to clearly demonstrate the relationships between IPMNs and pancreatic duct system;
- identifying high-risk stigmata or worrisome features is recommended in order to suggest the correct management;
- in case of IPMNs with high-risk stigmata, a surgical approach is needed, namely, for lesions with marked dilatation of the main pancreatic duct (≥1 cm) or showing internal solid enhancing components;
- if worrisome features are depicted on cross-sectional imaging modalities, EUS investigation is required. Confirmation of these worrisome features requires surgery. In their absence, a follow-up procedure by CT/MRI could be safely adopted, monitoring the development of malignant signs.

Finally, all imaging features should be related to clinical conditions of patients (age, comorbidities, and performance status) for a correct management of the disease.
